# Designing Uniformly Layered FeTiO_3_ Assemblies Consisting of Fine Nanoparticles Enabling High-Performance Quasi-Solid-State Sodium-Ion Capacitors

**DOI:** 10.3389/fchem.2020.00371

**Published:** 2020-05-27

**Authors:** Lei Liu, Zhongchen Zhao, Zhengqiang Hu, Xiangjun Lu, Shijia Zhang, Ling Huang, Yi Zheng, Hongsen Li

**Affiliations:** ^1^Key Laboratory of Functional Materials and Applications of Fujian Province, School of Material Science and Engineering, Xiamen University of Technology, Xiamen, China; ^2^Center for Marine Observation and Communications, College of Physics, Qingdao University, Qingdao, China

**Keywords:** FeTiO_3_, layered structure, sodium ion capacitor, quasi-solid-state, high performances

## Abstract

Sodium-ion capacitors (NICs) that have integrated the dual advantages of the high output of supercapacitors and the high energy density of batteries have stimulated growing attention for the next generation of practical electrochemical energy storage devices. The last years have seen the unprecedentedly rapid emergence of ilmenite materials, which present great promise in the realm of energy storage. However, NICs based on ilmenite materials have been scarcely researched so far. Instead, most of the current devices explored applied flammable liquid electrolytes, leading to a concern about unexpected leakage and potential safety problems. Herein, a quasi-solid-state NIC is constructed by employing the prepared uniformly layered FeTiO_3_ assemblies consisting of fine nanoparticles as anode and sodium ion conducting gel polymer as electrolyte. The resulting device delivers a high-energy-high-power density (79.8 Wh kg^−1^, 6,750 W kg^−1^), putting it among the state-of-the-art NICs. Furthermore, the assembled quasi-solid-state device also manifests long-term cycling stability over 2,000 cycles with a capacity retention ~80%. The uniformly layered FeTiO_3_ has great potential in developing low-cost and high-performance electrodes for the next generation of sodium and other metal ions-based energy storage devices.

**Graphical Abstract:**
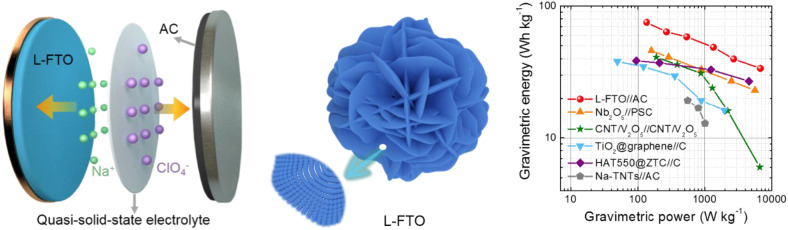
The high performance L-FTO//AC quasi-solid-state NIC device.

## Introduction

Electrochemical energy storage devices capable of high power and energy density are gaining widespread attention on account of the great industrial demands in the portable electronic, electrical vehicles, and smart grid markets (Dunn et al., 2011; El-Kady et al., 2015; Lu et al., 2017). As one of the widely researched and applied energy storage devices, metal-ion batteries (such as Li and Na-ion batteries) possess high energy density but suffer from a low power output and short life span. Supercapacitors (SCs) exhibit long cycle life as well as high power but their energy density is not desirable (Wang et al., 2016; Yu et al., 2017a,b; Li et al., 2018a; Fan et al., 2019; Zhao et al., 2019a). In recent years, the trade-off between the energy and power density of electrochemical energy storage mechanisms has been systematically studied, which essentially derives from the electron storage mechanism of electrodes (the slow ion deintercalation/intercalation in metal-ion batteries and formation of double-layer in SCs). Therefore, it is imminently desired to assemble a metal-ion hybrid capacitor with a capacitor-like high rate and battery-like high capacity (Aravindan et al., 2014; Li et al., 2016a,b, 2018b; Zhang et al., 2017; Li Y. et al., 2018; Ge et al., 2020). Furthermore, the present urgent concerns over the cost of lithium resources have forced attentions on to sodium-based energy storage devices, owing to the element abundance and high cost performance of sodium (Wang et al., 2015a,b; Zhao et al., 2019a).

Assembling sodium-ion capacitors (NICs) remains a great challenge due to the lack of appropriate battery-like anode materials that are able to combine both excellent rate capability and long cycling stability. In the past few years, great efforts have been made to find suitable anode materials. Transition metal oxides have been extensively studied as anodes for Na-ion batteries and NICs because of their low cost and high capacity. However, the options are quite limited due to the competition between conversion and insertion (Senguttuvan et al., 2013). The Ti-based oxide materials have been proven as a suitable alternative to the above-mentioned material owing to their low working potentials, environmental benignity, and high electrochemical performance. For example, Na_2_Ti_3_O_7_, Na_2_Ti_9_O_19_, and TiO_2_ are studied as ideal anodes for SIBs owing to their negligible structure deformation during the (de)insertion process of Na^+^ (Li et al., 2016a,c; Zhang et al., 2016; Bhat et al., 2017; Zhou et al., 2017). However, the irreversible phase transformation after multiple charge/discharge processes cause large volume deformation that restricts their wide application. In general, the effective modification strategies such as preparation of materials in microscale/nanoscale size, optimization of material crystallinity and structure, and construction of surface microstructure, have been widely used in these Ti-based materials. Li and co-workers have developed the Na_2_Ti_3_O_7_ nanotubes with layered crystalline structures, which delivered an enhanced electrochemical performance (Li et al., 2016a). As a set of semiconductor materials, the development of these Ti-based anode materials is also limited by the poor conductivity and low diffusion coefficient of Na^+^. Hwang and co-workers demonstrated that nanostructured TiO_2_ doped with a certain amount of fluorine could effectively improve the problem of sluggish kinetics of Na^+^ (Hwang et al., 2015). In addition to considering the effect of nanostructure and doping, considerable efforts have also been concentrated on the synergistic effect of the foreign metal ions for better electrochemical performance. Among the various metallic elements, Fe is selected to synthesize nanostructured FeTiO_3_ in consideration of the following factors: FeTiO_3_ with low toxicity has a rich reserve (over 680 million tons in the earth's crust), and low cost (no more than 350 USD/metric ton) (Yu et al., 2017a). Besides, among these Ti-based materials, FeTiO_3_ is an engaging member in ABO_3_-type structure to which the size of A (Fe) and B (Ti) are approximately identical. As a unique bimetal oxide, nanostructed FeTiO_3_ has been widely demonstrated in metal-ion batteries, catalytic activity, and gas sensors so far, however, its application in NICs is still absent. Furthermore, most of the NICs explored were based on the high flammability and volatility liquid organic electrolyte (such as NaClO_4_ in EC/DEC), which increases the concern about potential safety. Hence, the realizing of solid-state SICs employing a quasi-solid-state or solid-state electrolytes is desirable (Guo et al., 2018).

Herein, by means of a facile solvothermal reaction coupled with further calcination, we successfully designed and synthesized the uniformly layered FeTiO_3_ assembles (L-FTO), which was further served as the superior anode material for quasi-solid-state NICs. A kind of Na^+^ conducting gel polymer employing the P(VDF-HFP) membrane as the matrix was used as the electrolyte. Benefiting from the structure of uniformly layered FeTiO_3_ assembly consisting of fine nanoparticles, the L-FTO electrode exhibited superior electrochemical performances in SIBs (a capacity of 93.7 mA h g^−1^ at 10 A g^−1^, and a long-term lifetime of over 2,000 cycles). Moreover, with the L-FTO as the battery-type anode and active carbon (AC) as the supercapacitor-type cathode, the quasi-solid-state NICs delivered remarkably improved electrochemical performances with excellent rate capability and high-power-high-energy density (79.8 Wh kg^−1^, 6,750 W kg^−1^) as well as a long life span (~80% retention after 2,000 cycles at 5 A g^−1^).

## Experimental

### Synthesis of L-FTO

All of the raw materials were used as received without further purifications. L-FTO was fabricated by a solvothermal route. In a typical process, 0.9 mmol of Iron (III) chloride hexahydrate (FeCl_3_·6H_2_O, Sigma Aldrich) and 5 mmol urea were dispersed in 40 mL Ethylene glycol (EG) by vigorously stirring for 40 min under room temperature to form the transparent yellow solution. Afterward, 46 μL of Tetrabutyl titanate (TBOT, Sigma Aldrich) was added into the yellow solution under continuous stirring. After stirring for 15 min, the solution was then transferred into 60 mL Teflon-lined stainless-steel autoclave. The autoclave was kept at 160°C for 12 h. The precursor product was collected and washed with ethanol several times. Subsequently, the precursor product was dried at 60°C for 12 h in a vacuum. Finally, the L-FTO was obtained upon calcination at 600°C in an argon atmosphere for 10 h with a ramping rate of 2°C min^−1^.

### Synthesis of Sodium Ion Conducting Gel Polymer

The prepared porous P(VDF-HFP) membrane was synthesized according to the previous literature reported by Yang's group with minor modifications (Yang et al., 2015). Briefly, 7.5 g of P (VDF-HFP) powder was dispersed into the mixed solution of 42.8 mL of DMF and 1.5 mL of deionized water (DI) at 80°C. The resulting transparent solution was then evenly coated on a glass plate. After soaking in a water bath at 80°C for 30 min, the sol-gel coated on the glass plate formed a porous white homogeneous membrane. After that, the white membrane was dried under a vacuum at 100°C for 12 h. Finally, the dried P(VDF-HFP) membrane was cut into pieces of a particular size. To enable flexible quasi-solid-state sodium ion capacitors with high energy density, these membranes were immersed in an organic electrolyte [1 M sodium perchlorate (NaClO_4_) in a 1:1 mixture of ethylene carbonate (EC) and dimethyl carbonate (DEC)] over 12 h in a glove box(water content: <1 ppm, oxygen content: <1 ppm).

### Material Characterization

The samples were characterized using XRD (Bruker D8 advance) with a Cu Kα radiation at 40 kV and 30 mA, field-emission scanning electron microscopy (FESEM) (ZEISS, Sigma 300), and transmission electron microscopy (TEM) (JEOL, JEM-2100F). The X-ray photoelectron spectroscopy (XPS) measurements were performed by an ESCALAB250Xi system using a monochromatic Al Ka1 source. To reveal the carbon content of L-FTO, the thermogravimetric analysis (TGA, Netzsch STA449) was collected on a temperature ramp of 10°C min^−1^ under air flow.

### Electrochemical Measurement

Standard CR2032-type coin cells were assembled in an argon-filled glove box with the as-fabricated L-FTO as the working electrode. Half-cell configurations were assembled using synthesized sodium ion conducting gel polymer as the electrolyte, and Na-metal foil as counter and reference electrodes. The L-FTO anode was prepared by a slurry-coating process. The slurry consisted of 80 wt% active materials, 10 wt% conductive carbon black (Super P), and 10 wt% carboxyl methyl cellulose (CMC) dissolved in DI water. The slurry was then casted on copper foil. The thickness and areal loading density of the prepared L-FTO electrodes were ~13.2 μm and 0.9 mg cm^−2^, respectively. The AC cathode was prepared using the same procedure. The slurry was combined with 80 wt% active materials, 10 wt% Super P, and 10 wt% polyvinylidenediflouride (PVDF) dissolved in N-methylpyrrolidone (NMP), which was spread on aluminum foil. The average loading mass of the cathode material was ~4.4 mg cm^−2^. Electrochemical measurements of the quasi-solid-state NICs were tested using two-electrode cells at room temperature. The aforementioned FTO and AC electrodes were performed as the anode and cathode, respectively. The cell balance was achieved by setting the electrode mass ratio of cathode/anode at about 5:1. Both the L-FTO anode and the AC cathode were galvanostatically (0.1 A g^−1^) cycled a few times in half cells (i.e., vs. Na/Na^+^), which ending in a desodiated and sodiated condition, respectively. Then, the half cells were disassembled, and the electrodes were taken out and re-assembled into a full cell. Cyclic voltammetry (CV) curves were carried out by an electrochemical workstation (IVIUM technologies, Vetex. One. EIS) at 0.5 mV s^−1^. Galvanostatic charge/discharge experiments were performed at different current densities on a LAND battery test system (CT2001A).

The energy density (E) and power density (P) of NICs against the two electrodes in the device were calculated based on the total mass of the active materials using the following equations:

(1) $$\begin{array}{rcl} E & = & \int_{t1}^{t2}IV\;dt \end{array}$$

(2) $$\begin{array}{rcl} P & = & \frac{E}{t} \end{array}$$

where *E* (Wh kg^−1^) is energy density, *P* is power density (W kg^−1^), *I* is the constant current density (A g^−1^), V is the voltage, *t1, t2* is the start time and end time in the discharge process, and t is discharge time (Li et al., 2016a).

## Results and Discussions

**Scheme 1:**
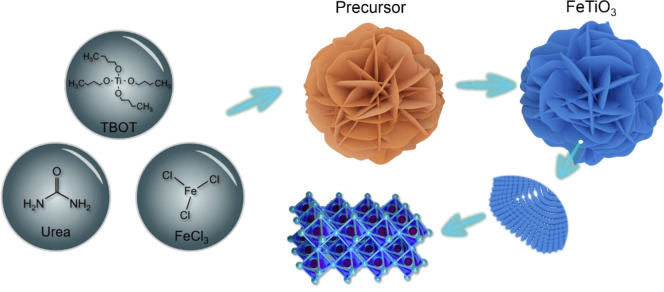
Schematic illustration for preparation of the L-FTO.

**Figure 1:**
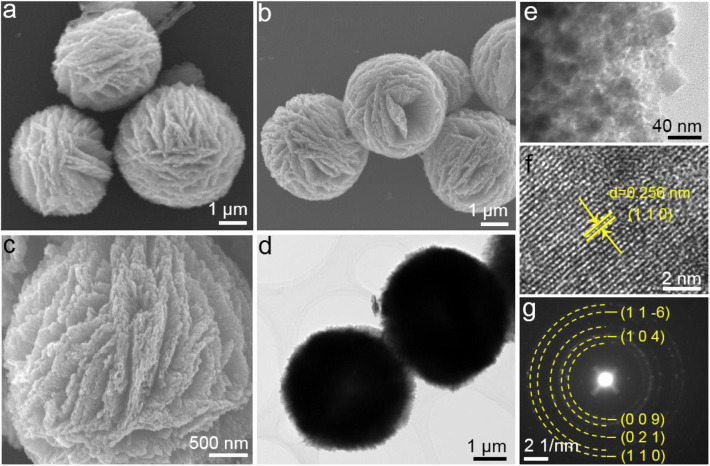
**(A–B)** FESEM images, **(D,E)** TEM images, **(F)** HRTEM image, **(G)** the corresponding SAED pattern of the obtained L-FTO.

The fabrication schematic is illustrated in detail in Scheme 1. Briefly, the homogeneous precursors were firstly synthesized by a one-pot solvothermal method using FeCl_3_·6H_2_O as the iron source and tetrabutyl titanate (TBOT) as the titanium source. In the subsequent step, the precursor was further converted into uniformly-layered FeTiO_3_ assembles with high crystallinity through calcination at 600°C for 10 h in Ar atmosphere. The general morphology and detailed nanostructure of the L-FTO were investigated by employing Field-emission scanning electron microscopy (FESEM) and transmission electron microscopy (TEM), respectively. The unique multi-layer structure, homogeneity, and monodispersed of the synthesized L-FTO and precursor are shown in Figure 1, Figure S1, respectively. It can be seen that the precursor was assembled with nanosheets, exhibiting a diameter of ~4 μm (Figure S1). After annealing at 600°C in Ar, the precursor was fully transformed to uniformly layered FeTiO_3_ without changes to the multilevel structure (Figures 1A–C). Interestingly, Figure 1C displays well-defined layered FeTiO_3_ assembles, which can be observed that the 2D asperous nanosheets are constructed by fine nanoparticles. This unique structure could provide short transportation paths of ions and abundant active sites for ion/electron exchange between anode and electrolyte, which is favorable to achieving high rate performance. The detailed microstructure and crystalline features of L-FTO were investigated by TEM and HRTEM (Figures 1D–G). In accordance with that which was observed in the FESEM images, the TEM images shows that the L-FTO structure consisted of numerous fine nanoparticles (Figure 1E). Additionally, the lattice spacing of L-FTO in representative HRTEM (Figure 1F) is 0.256 nm, corresponding to the interplanar distance of (110) planes of L-FTO phase (JCPDS No. 75-1212). The select area electron diffraction (SAED) pattern shown in Figure 1G exhibits that five rings are consistent with (11–6), (104), (009), (021), and (110) planes of rhombohedral L-FTO.

**Figure 2:**
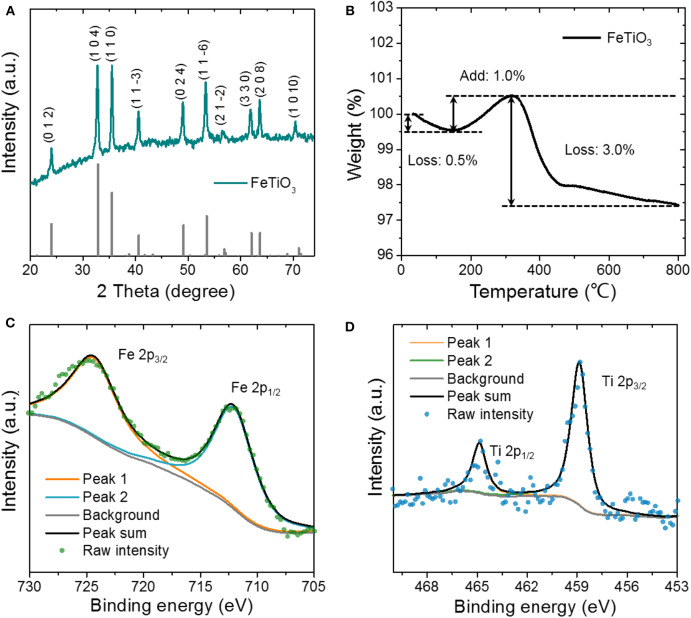
**(A)** XRD pattern, **(B)** TG curve, **(C)** High-resolution Fe 2p, and **(D)** Ti 2p XPS spectra of the L-FTO.

The phase purity and crystallographic structure of the L-FTO were confirmed by X-ray diffusion (XRD) analysis. As shown in Figure 2A, all characteristic peaks at 23.9, 32.8, 35.5, 40.6, 49.1, 53.6, 56.9, 62.2, and 63.7° are observed in the XRD pattern of samples, which are in good agreement with (012), (104), (110), (113), (024), (11–6), (018), (124), and (300) crystal planes of rhombohedral FeTiO_3_ standard card (JCPDS 75-1212), indicating high purity of product (Yu et al., 2017a). The carbon content was evaluated by thermal gravimetric analysis (TGA) as shown in Figure 2B. The initial weight increase between 150 and 330°C can be attributed to the oxidation of L-FTO to TiO_2_ and Fe_2_O_3_ in the air (Xiong et al., 2011; Yu et al., 2017a). When the sample was heated up to 330°C in the air, the weight loss of ~3% that occurred between 330 and 450°C can be ascribed to the combustion of carbon in the air (Yu et al., 2017a):

(3) $$\begin{array}{l} \frac{1-a}{152}FeTiO_{3}+\frac{a}{12}C\vec{air}\;{\frac{1-a}{2^{*}152}}^{*}Fe_{2}O_{3}\\ \;+\frac{1-a}{152}*TiO_{2}+\frac{a}{12}CO_{2}↑ \end{array}$$

Where *a* corresponds to the carbon content of the sample. The results reveal that the weight of carbon in the hybrid is quite small, about 7.5 wt%.

The chemical composition and surface chemical bonding state of the L-FTO were further determined via X-ray photoelectron spectroscopy (XPS) (Figures 2C,D, Figure S2). The high-resolution XPS spectrum of Fe 2p showing the main Fe 2p_3/2_ peak position is observed at 711.9 eV, corresponding to the elements in the oxidation states Fe^2+^. The centroid of the main Ti 2p_3/2_ is found at 458.8 eV, confirming the Ti^4+^ state of Ti. A single feature from the O 1 s binding energy is 533.7 eV, suggesting that the only O^2−^ exists in the sample (Ding et al., 2018). The XRD pattern of AC used in this work displays two broad peaks concentrated at 23.2 and 43.7°, which correspond to (002) and (100) planes of graphite (Figure S3) (Wang et al., 2015a). Figure S4 shows the FESEM image of the synthesized porous P(VDF-HFP) membrane. It can be clearly observed that abundant pores were uniformly distributed on the surface of the membrane with sizes of 1–2 μm. Taking this advantage, the P(VDF-HFP) membrane can effectively absorb and maintain the organic electrolyte. After immersing the P(VDF-HFP) membrane in the electrolyte, the liquid organic electrolyte can be trapped in the pores of the polymer matrix, preventing leakage. When enough electrolyte was soaked in the matrix, the mixture was transformed into membrane between liquid and solid states, that is, the quasi-solid-state electrolyte. According to previously reported literature, this type of Na^+^ conducting gel polymer electrolyte possessing a high ion conductivity of ~0.6 mS cm^−1^ could be suitable for application as an ideal electrolyte for sodium ion batteries and capacitors (Yang et al., 2015).

**Figure 3:**
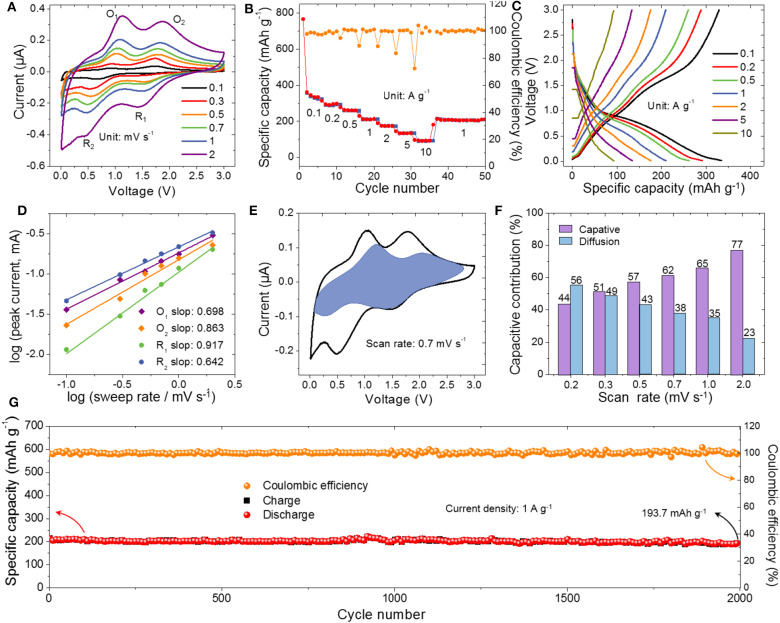
Electrochemical characteristics of the L-FTO in half cells. **(A)** CV curves of L-FTO electrode at various scan rates from 0.1 to 2 mV s^−1^. **(B)** The corresponding voltage profiles and rate capability **(C)** at different current densities from 0.1 to 10 A g^−1^. **(D)** Plot of log (i) vs. log (ν) of anodic/cathodic peaks from CV scans and b-value determination lines. **(E)** Separation of the pseudocapacitive and diffusion currents at a scan rate of 0.7 mV s^−1^. **(F)** Pseudocapacitive contribution ratio at different scan rates from 0.2 to 2 mV s^−1^. **(G)** The cycling performance with coulombic efficiency over 2,000 cycles at 1 A g^−1^.

The Na^+^-ion storage behaviors of the L-FTO anode and AC were evaluated by assembling 2032 coin-type cells with a metallic Na counter electrode and Na^+^ conducting gel polymer electrolyte. Figure 3A shows the CV curves of the L-FTO in the voltage of 0.01–3.0 V vs. Na^+^/Na at various scan rates from 0.1 to 2 mV s^−1^. Two pairs of well-defined redox peaks can be observed, which correspond to the Fe^2+^/Fe^0^ and Ti^4+^/Ti^3+^ redox couples, respectively (Yu et al., 2017a). Importantly, the CV curve maintained a similar shape even at a scan rate of 2 mV s^−1^, indicating the efficient diffusion of Na^+^-ions into the L-FTO anode. Figure 3B exhibits the galvanostatic discharge/charge performances of L-FTO between the 0.01–3.0 V at various current densities. The initial discharge and charge specific capacities at 0.1 A g^−1^ are 766.3 and 358.3 mAh g^−1^, respectively. The capacity loss generally comes from the irreversible decomposition of electrolyte and the formation of Na_2_O, Ti, and solid-electrolyte interface (SEI) layer (Narendra et al., 2018). After cycling a few cycles, the discharge/charge specific capacities of L-FTO quickly stabilizes, indicating that the electrochemical Na^+^-ions (de)insertion reactions are highly reversible and stable in the electrode. As shown in Figure 3C, the high specific capacities of 334.2, 296.3, 261.4, 210.3, 174.7, 134.5, and 93.7 mAh g^−1^ at the current density of 0.1, 0.2, 0.5, 1, 2, 5, and 10 A g^−1^ are achieved, respectively. In addition, the discharge capacity could recover to 214.5 mAh g^−1^ when the current density was adjusted from 10 to 1 A g^−1^. The high rate properties could be attributed to the hierarchical nanostructured morphology and stable crystal structure of L-FTO, demonstrating that the L-FTO is a promising battery-type electrode for the SICs. To make out the Na^+^ storage kinetics of the L-FTO, the redox pseudocapacitance-like contribution was analyzed as depicted in Figures 3D–F. The peak current obeyed a power law relationship with the scan rate, according to Equation (4).

(4) $$\begin{array}{r} i=av^{b} \end{array}$$

where *v* is the sweep rate, *i* is the peak current, and *a* and *b* are adjustable parameters. The value of *b* could be obtained by plotting the log(i)-log(v) curves. When *b* is roughly equal to 0.5, the ionic diffusion could control the electrochemical reaction. Conversely, when the b value is approximately equal to 1, the process is mainly determined by the surface capacitive effect. The plots log (*i*) vs. log (*v*) for cathodic and anodic peaks are presented in Figure 3D. The *b* values were calculated to be 0.917, 0.698 at R_1_ and O_1_, and 0.642 and 0.863 at R_2_ and O_2_ correspondingly, suggesting that the charge storage behavior was partially pseudocapacitive. Moreover, the level of capacitive-controlled charge storage at different sweep rates were obtained according to Dunn's method. The total behavior can be distinguished into different mechanisms at settled potential by Equation (5).

(5) $$\begin{array}{rcl} i\left(V\right) & = & k_{1}v+k_{2}v^{1/2} \end{array}$$

(6) $$\begin{array}{rcl} i\left(V\right)/v^{1/2} & = & k_{1}v^{1/2}+k_{2} \end{array}$$

where k is a constant. The values of k_1_ and k_2_ could be determined by using Equation (6) (Zhao et al., 2019b). For instance, the capacitive contribution ratio reached 62% at a scan rate of 0.7 mV s^−1^ (Figure 3E). In addition, the capacitive contribution of L-FTO gradually increases with the increase of the scan rate from 0.1 to 2.0 mV s^−1^, which is beneficial to high rate capacity and long term cyclability for SICs (Figure 3F). The cyclic performance at a representative current density of 1 A g^−1^ is shown in Figure 3G. An impressive value of nearly 98% capacity retention (186 mAh g^−1^) with a high coulombic efficiency (above 99%) is available after 2,000 cycles.

The electrochemical performance of the AC electrode was also evaluated by CV and cycle analysis in the voltage range of 1.5–4.2 V (Figures S5, S6). A near-rectangular shape is exhibited in the CV curve at the scan rate of 0.5 mV s^−1^, suggesting typical capacitive behavior (Figure S5). The rate performance of AC was investigated (Figure S6), and we found that the AC electrode displayed a high capacity (~50 mAh g^−1^) at the current density of 10 A g^−1^. Presented in Figure S7 was the long-term cycling performance of AC at a current density of 1 A g^−1^, exhibiting no noticeable capacitance decay over 2,000 cycles.

**Figure 4:**
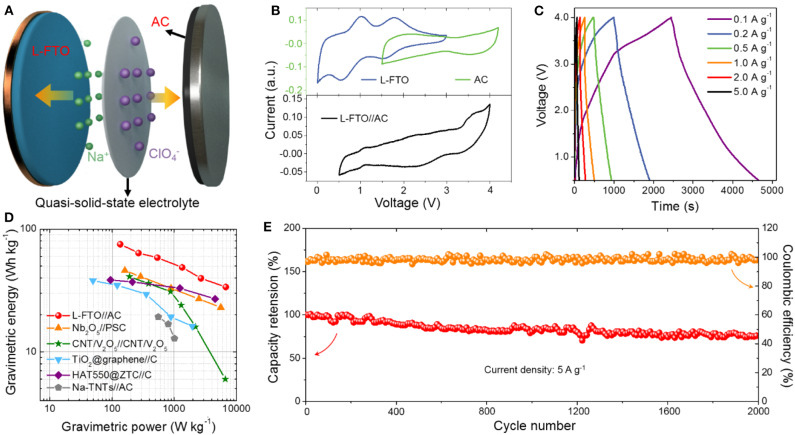
**(A)** Schematic illustration of the L-FTO//AC quasi-solid-state NIC device. **(B)** CV profiles of L-FTO and AC in half cells (top) and full cell of the quasi-solid-state NIC device (bottom) at scan rate of 0.5 mV s^−1^. **(C)** Typical galvanostatic charge-discharge curves at different current densities. **(D)** Ragon-plot of the L-FTO//AC quasi-solid-state NIC device in comparison with other reported results (Chen et al., 2011; Yin et al., 2012; Li et al., 2016b; Le et al., 2017; Yan et al., 2019). **(E)** Long-term cycling performance of the L-FTO//AC quasi-solid-state NIC device (at 5 A g^−1^).

To further demonstrate the potential of L-FTO in quasi-solid-state SICs, a prototype full cell was fabricated by utilizing L-FTO as the anode electrode, AC as the cathode electrode, and the sodium ion conducting gel polymer as the electrolyte (Figure 4A). The L-FTO electrode and AC electrode possess stable potential windows of 0.01–3 V and 1.5–4.2 V, respectively (Top curves in Figure 4B). To balance the charge between the anode and cathode, the CV curves of the assembled L-FTO//AC quasi-solid-state SICs collected at 0.5 mV s^−1^ with voltage window from 1.5 to 4 V are shown in Figure 4B. Additionally, all the charge-discharge curves at current densities from 0.1 to 5 A g^−1^ show an asymmetric triangular shape, suggesting a fast kinetics character of the L-FTO//AC quasi-solid-state SICs. As shown in Figure 4C, high energy density at different current densities could be realized in the assembled L-FTO//AC quasi-solid-state SICs with anode/cathode mass ration of 1/5. To be specific, it exhibited the high energy-density of 79.8 Wh kg^−1^ (at a power-density of 135 W kg^−1^) and it still displayed an impressive energy-density of 35.7 Wh kg^−1^ at the high-power density of 6,750 Wh kg^−1^, which is quite attractive for practical application. Additionally, Figure 4D shows the ragone plots of our L-FTO//AC quasi-solid-state SICs, and some previous reports about NICs were also added for-comparison including CNT/V_2_O_5_// CNT/V_2_O_5_, TiO_2_@graphene//C, Nb_2_O_5_//PSC, and so on. The energy density of this work is superior to that of recently reported NIC devices. Furthermore, the cycling stability of the L-FTO//AC quasi-solid-state devices were characterized at a current density of 5 A g^−1^, and the device could retain columbic efficiency of ~100% up to 2,000 cycles with capacity retention of about 80% (Figure 4E).

## Conclusion

In summary, L-FTO was synthesized by a facile solvothermal reaction coupled with a further calcination route and introduced for the first time as the anode candidate for a high-performance quasi-solid-state NIC. The well-defined microstructure can effectively buffer structural deformation, boost the Na^+^-ion/electron transport, and increase the interfacial contact between electrolyte and electrode, thereby resulting in significant enhanced kinetics. Accordingly, L-FTO deliver remarkable sodium ion storage performance as a suitable anode for half-cells, endowed with preferable rate capability, high specific capacities, and long cyclic stability, demonstrating their potential application as an anode material for high-power-high-energy NICs. Attributing to the advantages of properties and structures of the L-FTO, the assembled L-FTO//AC quasi-solid-state NIC delivered a maximum energy density of 79.8 Wh kg^−1^ and power density of 6,750 W kg^−1^, along with an ultralong life span in the voltage window of 0.5–4.0 V. With optimization, the resulting L-FTO//AC quasi-solid-state NICs with superior electrochemical performance could be a promising competitor for electrochemical energy storage.

## Data Availability Statement

The datasets generated for this study are available on request to the corresponding author.

## Author Contributions

All authors listed have made a substantial, direct and intellectual contribution to the work, and approved it for publication.

## Conflict of Interest

The authors declare that the research was conducted in the absence of any commercial or financial relationships that could be construed as a potential conflict of interest.
