# Supplementary material for: Designing Uniformly Layered FeTiO3 Assemblies Consisting of Fine Nanoparticles Enabling High-Performance Quasi-Solid-State Sodium-Ion Capacitors
Source: Front Chem. 2020 May 27;8:371. doi: 10.3389/fchem.2020.00371 (PMC7267067; doi:10.3389/fchem.2020.00371)
Supplement: Supplementary file 1 [file Data_Sheet_1.docx]

Supplementary Material

Designing uniformly layered FeTiO_3_ assemblies consisting of fine nanoparticles enabling high-performance quasi-solid-state sodium-ion capacitors

Lei Liu^1#^, Zhongchen Zhao^2#^, Zhengqiang Hu^2^, Xiangjun Lu^1*^, Shijia Zhang^2^, Ling Huang^2^, Yi Zheng^2^, Hongsen Li^2*^

^1^ Key Laboratory of Functional Materials and Applications of Fujian Province, School of Material Science and Engineering, Xiamen University of Technology, Xiamen, 361024, China

^2^ College of Physics, Center for Marine Observation and Communications, Qingdao University, Qingdao, 266071, China

*** Correspondence:**Hongsen Li, [hsli@qdu.edu.cn](mailto:hsli@qdu.edu.cn)

## Xiangjun Lu, luxiangjun0531@163.com

## Supplementary Figure


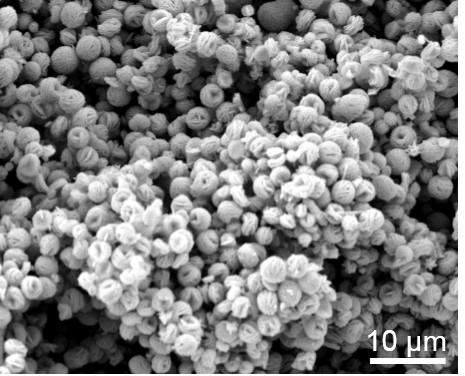


**Figure S1.** SEM image of the precursors.

## Supplementary Figure


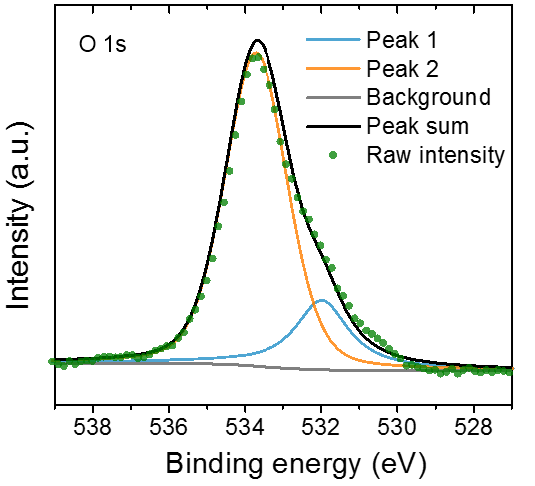


**Figure S2**. Detailed XPS spectra of O 1s.

## Supplementary Figure


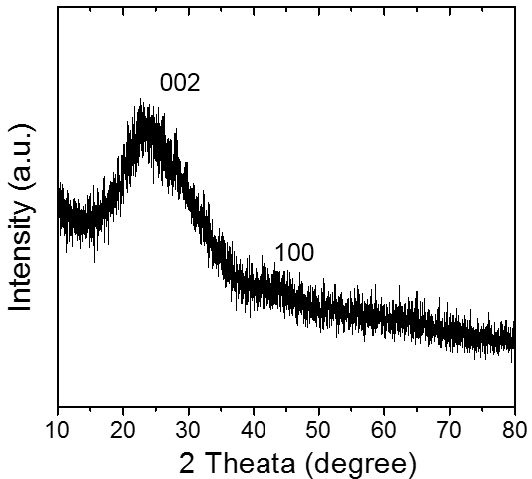


**Figure S3**. XRD pattern of AC.

## Supplementary Figure


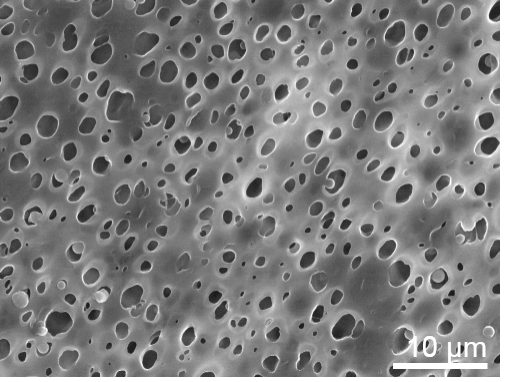


**Figure S4**. FESEM image of the prepared porous P(VDF-HFP) membrane.

## Supplementary Figure

**Figure S5**. CV curve of AC at scan rate of 0.5 mV s^-1^.


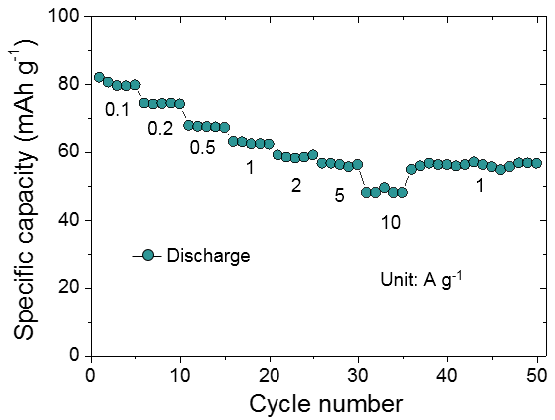


**Figure S6.** The corresponding rate capability of AC at different current densities from 0.1 to 10 A g^-1^.

## Supplementary Figure

**Figure S7**. Cycling performance of AC at a current rate of 1 A g^-1^ for 2000 cycles.
